# Oral health-related quality of life in 4–16-year-olds with and without juvenile idiopathic arthritis

**DOI:** 10.1186/s12903-022-02400-1

**Published:** 2022-09-06

**Authors:** Elisabeth G. Gil, Marit S. Skeie, Josefine Halbig, Birgitta Jönsson, Stein Atle Lie, Marite Rygg, Johannes Fischer, Annika Rosén, Athanasia Bletsa, Keijo Luukko, Xie-Qi Shi, Paula Frid, Lena Cetrelli, Karin Tylleskär, Karen Rosendahl, Anne N. Åstrøm

**Affiliations:** 1 Department of Clinical Dentistry, University of Bergen, Bergen, Norway; 2 Center for Oral Health Services and Research, TkMidt, Trondheim, Norway; 3 Public Dental Health Competence Centre of Northern Norway (TkNN), Tromsø, Norway; 4 Department of Clinical Medicine, The Arctic University of Norway, Tromsø, Norway; 5 Department of Periodontology, Institute of Odontology, University of Gothenburg, Gothenburg, Sweden; 6 Department of Clinical and Molecular Medicine, Norwegian University of Science and Technology (NTNU), Trondheim, Norway; 7 Department of Pediatrics, St. Olavs Hospital, Trondheim, Norway; 8 Department of Oral and Maxillofacial Surgery, Haukeland University Hospital, Bergen, Norway; 9 Oral Health Centre of Expertise in Western Norway, Vestland, Norway; 10 Department of Oral Maxillofacial Radiology, Faculty of Odontology, Malmö University, Malmö, Sweden; 11 Department of Otorhinolaryngology, Division of Oral and Maxillofacial Surgery, University Hospital of North Norway, Tromsø, Norway; 12 Department of Pediatrics, Haukeland University Hospital, Bergen, Norway; 13 Department of Radiology, University Hospital of North Norway, Tromsø, Norway

**Keywords:** Adolescent, Child, Quality of life, Dental caries, Oral health, Juvenile idiopathic arthritis

## Abstract

**Background:**

Few studies have investigated oral health-related quality of life (OHRQoL) in young individuals with juvenile idiopathic arthritis **(**JIA). Aims were to investigate whether OHRQoL differs between children and adolescents with JIA compared to controls without JIA, while adjusting for socio-demographic-, behavioral- and oral health-related covariates. Furthermore, to explore whether socio-behavioral and oral health-related covariates of OHRQoL vary according to group affiliation and finally, specifically for individuals with JIA, to investigate whether disease-specific features associate with OHRQoL. We hypothesized that participants with JIA have poorer OHRQoL compared to participants without JIA.

**Methods:**

In this comparative cross-sectional study participants with JIA (n = 224) were matched to controls without JIA (n = 224). OHRQoL was assessed according to Early Childhood Oral Health Impact Scale (ECOHIS) (4–11-years-olds) and the child version of Oral Impacts on Daily Performances (Child-OIDP) (12–16-years-olds). JIA-specific characteristics were assessed by pediatric rheumatologists and socio-demographic, behavioral and self-reported oral health information collected by questionnaires. Index teeth were examined for caries by calibrated dentists. Multiple variable analyses were performed using logistic regression, reporting odds ratio (OR) and 95% confidence interval (CI). Two-way interactions were tested between group affiliation and the socio-behavioral- and oral health-related variables on the respective outcome variables.

**Results:**

In total, 96 participants with JIA and 98 controls were evaluated according to ECOHIS, corresponding numbers for Child-OIDP was 125 and 124. Group affiliation was not associated with impaired ECOHIS or Child-OIDP in adjusted analyses (OR = 1.95, 95% CI 0.94–4.04 and OR = 0.99, 95% CI 0.46–2.17, respectively). Female adolescents with JIA were more likely than males to report oral impacts according to Child-OIDP. Continued activity or flare was found to adversely affect Child-OIDP, also self-reported outcome measures in JIA associated with Child-OIDP.

**Conclusions:**

This study did not provide consistent evidence to confirm the hypothesis that children and adolescents with JIA are more likely to have impaired OHRQoL compared to their peers without JIA. However, female adolescents with JIA were more likely than males to report impacts on OHRQoL. Furthermore, within the JIA group, adolescents with continued disease activity, flare or reporting pain, physical disability, had higher risk than their counterparts of impaired OHRQoL.

**Supplementary Information:**

The online version contains supplementary material available at 10.1186/s12903-022-02400-1.

## Background

As the most common chronic rheumatic disease in children, juvenile idiopathic arthritis (JIA) represents a complex heterogeneous group of arthritis and might constitute an important cause of disability and reduced quality of life [1, 2]. Pooled estimates of incidence and prevalence rates for Caucasians are 8.3/100,000 and 32.6/100,000, respectively, but estimates vary greatly across countries [3]. For most children JIA is a chronic, often life-long, disease. Objectives of disease management is remission, minimizing comorbidities and harmful side-effects of medication, and achieving best possible function, growth and development, quality of life, and social involvement [4].

Several manifestations of rheumatic diseases are observed in the oral cavity such as mucosal lesions, reduced salivary flow and inflammation in periodontal tissues [5]. Previous reviews have demonstrated poor oral health indicators in children with JIA [6, 7]. A recent systematic review and meta-analysis by our research team focusing on oral disease and problems among children and adolescents with JIA, revealed, however, that dental caries in young individuals with JIA was comparable to that of the general population, whereas periodontal diseases and temporomandibular disorder (TMD) were estimated to be more prevalent, compared to individuals without JIA [8].

According to the definition of the World Dental Federation [9, 10], reflecting the biopsychosocial view of health addressed by the World Health Organization [11], oral health encompasses “the ability to speak, smile, taste, touch, chew, swallow, and convey a range of emotions through facial expressions with confidence and without pain, discomfort, and disease of the craniofacial complex”. To capture the different dimensions of oral health, oral health-related quality of life (OHRQoL) measurements are conducive, as conventional clinical oral indices alone are insufficient to assess the multifaceted nature of oral health. Generic OHRQoL instruments have been developed to measure physical- and psycho-social consequences of various oral diseases and problems, whereas condition specific OHRQoL measures capture subtle variance in specific oral conditions [12]. Measuring OHRQoL in children are challenging due to continuous dental, facial and cognitive development [13]. Thus, age-dependent generic child OHRQoL indices have been developed for self- or proxy-reporting of children’s OHRQoL [12], such as the Child Oral Health-related Quality of Life measure (COHQoL) [14–16], the child version of Oral Impacts on Daily Performances (Child-OIDP) [17], and the Early Childhood Oral Health Impact Scale (ECOHIS) [18]. The latter specifically developed for younger children. Only ECOHIS and Child-OIDP have been validated in the context of a Norwegian child and adolescent population [19]. Although these indices have been used to evaluate OHRQoL in children and adolescents with chronic diseases [20, 21], studies investigating OHRQoL in children and adolescents with JIA are scarce [22–26].

Isola et al. [23] reported that individuals with temporomandibular joint (TMJ) arthritis had poorer OHRQoL compared to individuals with JIA without TMJ arthritis and controls, using the Child Perception Questionnaire (CPQ_11-14_), a component of the inventory COHQoL. Using CPQ_11-14,_ Polizzi et al. [25] found JIA patients with periodontitis to have poorer OHRQoL, compared to JIA patients without periodontitis and controls. Santos et al. [22] observed no difference in impaired OHRQoL in individuals with JIA compared to controls using the Parental-Caregiver Perceptions Questionnaire, another component of the COHQoL inventory. The Psychosocial Impact of Dental Aesthetics Questionnaire has also been used among adolescents with JIA and controls of same age, indicating that adolescents with JIA were less concerned by dental aesthetics than controls [24]. Furthermore, Rahimi et al. [26] documented self-reported orofacial symptoms and dysfunction to be frequent in adolescents with JIA and by using CPQ_11-14_ they found orofacial symptoms to have a negative impact on OHRQoL.

Evidently, demographic- and socio-economic characteristics in addition to clinical indicators of oral health, play a prominent role as independent determinants of OHRQoL [27–29]. However, none of the previous studies assessing OHRQoL in children and adolescents with JIA [22–26] have included socio-economic characteristic of the participants as important covariates of OHRQoL. Thus, knowledge of the impact of social-economic characteristics on OHRQoL among young individuals with JIA and whether the impact of those characteristics differs between individuals with and without JIA is quite limited. Hence, high-quality research focusing on OHRQoL in children and adolescents with JIA is in demand [8]. Such studies are important as they facilitate comprehension of the relationship between oral health and general health [13].

The aims of this study were to investigate whether OHRQoL, assessed by the ECOHIS and Child-OIDP scale, differs between children and adolescents with JIA compared to controls without JIA, while adjusting for socio-demographic-, behavioral- and oral health-related covariates. Furthermore, to explore whether socio-behavioral and oral health-related covariates of OHRQoL vary according to group affiliation and finally, specifically for individuals with JIA, to investigate whether disease-specific features associate with OHRQoL. We hypothesized that participants with JIA have poorer OHRQoL compared to participants without JIA.

## Methods

### Study design and participants

NorJIA^1^ is a prospective longitudinal multicenter study that contributes baseline data to the present comparative cross-sectional study. Baseline data on dental caries have recently been published [30], whereby sample size calculation and calibration are presented. A detailed description of sample size calculation (according to caries estimates) and calibration are presented in Additional files 1 and 2, respectively. Young individuals (4–16 years old) with JIA, diagnosed according to the criteria specified by the International League of Associations for Rheumatology (ILAR) [31] were invited to participate. The only exclusion criterion was the lack of written informed consent. Baseline data were collected between April 2015 and August 2018.

Specialists in pediatrics at three out of total four university hospitals, widely distributed across Norway (western, central, and northern Norway), were responsible for the enrollment of children and adolescents with JIA. After a thorough medical examination the participants were referred for an oral examination at the corresponding Oral Health Centre of Expertise and matched 1:1 with controls based on sex, age, center site, and mothers’ country of origin (western or non-western). The controls were without JIA and underwent an oral examination at one of seven different Public Dental Service clinics, representing both rural and urban communities [30]. The controls’ appointment was coordinated with a planned regular oral health check, and as incentive for participation, two cinema tickets were provided. The term group affiliation in this article reflects participants with JIA or controls.

### Oral health questionnaires

Self-administered questionnaires provided socio-demographic, behavioral and self-reported oral health information [30]. Socio-demographic variables included educational level of caregivers, number of caregivers in the household and mother’s country of origin. Behavioral variables consisted of toothbrushing and tooth flossing frequency, while self-reported oral health indicators were gingival bleeding during toothbrushing and pain or discomfort during toothbrushing. Moreover, evaluation of self-reported oral health and satisfaction with appearance of teeth (global measures) were collected (for the participants ≥ 12 years the global oral health measures were assessed by an interview). The coding of these self-reported variables is shown in Additional file 3: Table S1.

### ECOHIS

A validated Norwegian version of ECOHIS [19], originally developed by Pahel et al. [18], was used to evaluate caregivers’ perception of the OHRQoL of their 4–11 years-old children and their families with reference to the child’s entire lifetime experience of oral diseases and dental treatment. ECOHIS consists of thirteen items, composing the child impact section (first nine items) and the family impact section (last four items). Each item, originally assessed in terms of never = 0 to very often = 5, was dichotomized (0 = not affected, including the original category 0 and 1 = affected, including the original categories 1–5) and dummy variables were summarized into the Child impact- and Family impact scores. The ECOHIS total score was calculated by adding the Child impact and Family impact scores. Participants having two or more items of the ECOHIS unanswered were excluded from the analysis. The response category “I don’t know” were coded as missing and not considered in the analyses. Variables and response categories as originally coded and as re-coded for analyses are shown in Additional file 4: Table S1.

### Child-OIDP

Among participants 12–16 years, OHRQoL was measured by interview, using the 8-item Child-OIDP frequency inventory. The OIDP inventory was initially constructed for adults [32] and later modified for children [17]. This index considers difficulty in performing eight daily activities (eating, speaking, cleaning teeth, smiling-laughing-and showing teeth without embarrassment, sleeping and relaxing, emotional balance, social contact, schoolwork) due to problems with mouth or teeth, during the past 3 months. Each of the eight items, originally assessed in terms of never = 0 to every day/almost every day = 3, was dichotomized (0 = not affected, including the original category 0 and 1 = affected, including the original categories 1–3) and the dummy variables were summarized, forming the Child-OIDP simple count (SC) score. The Child OIDP SC score was dichotomized into 0 = no impacts and 1 = 1–8 impacts. Participants with two or more items of the Child-OIDP unanswered were excluded from the analysis. Variables and response categories as originally coded and as re-coded for analyses are shown in Additional file 5: Table S1.

### Medical examinations of the participants with JIA

The participants with JIA were examined by experienced pediatric rheumatologists and the included background variables in this sub-study were JIA category according to the ILAR classification criteria [31], age at JIA onset, disease duration, medication, activity/remission status, physician’s global assessment of disease activity visual analogue scale (MDgloVAS) [33], patient/parent-reported pain intensity visual analogue scale (VAS pain) [33], patient/parent-reported global assessment of overall well-being visual analogue scale (PRgloVAS) [33]. All visual analogue scales were measured on a 21-numbered circle VAS (0 = minimal impact, 10 = maximal impact), and reported by the parent if the child were below 9 years, otherwise by the patient. Disability was reported with the Childhood Health Assessment Questionnaire (CHAQ) (0 = no disability, 3 = maximal disability) [34]. The disease-specific clinical background variables are described in detail in Additional file 6 [30] and the coding of these variables is shown in Additional file 7: Table S1.

### Oral examination of all participants

The oral assessment was performed by calibrated dentists (n = 5) [30]. For this sub-study, the examination was restricted to caries in the primary second molars in the youngest age group [4–9-year-olds] and in permanent first molars in the oldest age group [10–16-year-olds]. A detailed 5-graded diagnostic tool was applied for decayed lesions, in which grades 1–2 represented enamel lesions and grades 3–5 dentin lesions [35]. Filled surfaces were also reported. Missing teeth were not included in this sub-study as very few teeth (primary teeth: n = 5) were extracted or indicated for extraction due to caries [30]. The caries examination consisted of both visual inspection and bitewing (BW) radiographs. BW was not taken if intermolar contact was lacking, the participants were younger than 5 years or in case of fixed orthodontic appliances when only occlusal surfaces were examined. As a background variable, caries was dichotomized as presence (d_1-5_f/D_1-5_F > 0) or absence (d_1-5_f/D_1-5_F = 0) of caries.

### Statistical methods

SPSS version 25.0 (IBM Corp. Released 2013, IBM SPSS Statistics for Windows, Armonk NY: IBM Corp) and STATA version 16 (Stata Corp LP, College Station, TX) were used for data analysis. Linear weighted Cohen's kappa was used to evaluate inter- and intra-rater reliability for the caries measurements. Mean and standard deviations (SD) were used to describe continuous demographic variables. Categorical variables were compared between individuals with JIA and controls by Cross tabulation and Chi-squared tests. Logistic regression analyses were applied with ECOHIS total score and Child-OIDP SC score as binary outcome measures reporting odds ratio (OR) and 95% confidence interval (CI). Negative binomial regression was implemented as a supplementary analysis with ECOHIS total score and Child-OIDP SC score as count variables reporting incidence rate ratios (IRR) with 95% CI. The multiple variable regression analyses included the main exposure variable, group affiliation, adjusted for covariates in terms of socio-behavioral- and clinical oral health-related variables that were statistically significantly associated with group affiliation and/or the respective OHRQoL outcomes in the unadjusted analysis. The adjusted regression analyses specifically for participants with JIA, included the covariates age, gender and parental educational level, and the JIA-specific variables were adjusted separately. McFadden's R^2^ was applied as a measure for the goodness of fit of the logistic regression models. The JIA categories, systemic arthritis (n = 7) and undifferentiated arthritis (n = 31) were not included in the statistical analysis. Internal consistency reliability of the OHRQoL inventories was assessed using Cronbach’s alpha. Discriminant validity was assessed by comparing the OHRQoL inventories with global measures of oral health. Two-way interactions were tested between group affiliation and the socio-behavioral- and oral health-related variables on ECOHIS and Child-OIDP. *P*-values less than 0.05 were considered statistically significant.

### Ethical approval

The regional ethics committee (2012/542/REC) approved the study. Approval was also obtained by leaders of different County Dental Health Authorities, at different Oral Health Centre of Expertise, and at the three pediatric departments at the university hospitals. Written informed consent was signed prior to participation. The NorJIA study is registered at ClinicalTrials.gov (No: NCT03904459, 05.04.2019).

## Results

### Sample characteristics

**Fig. 1:**
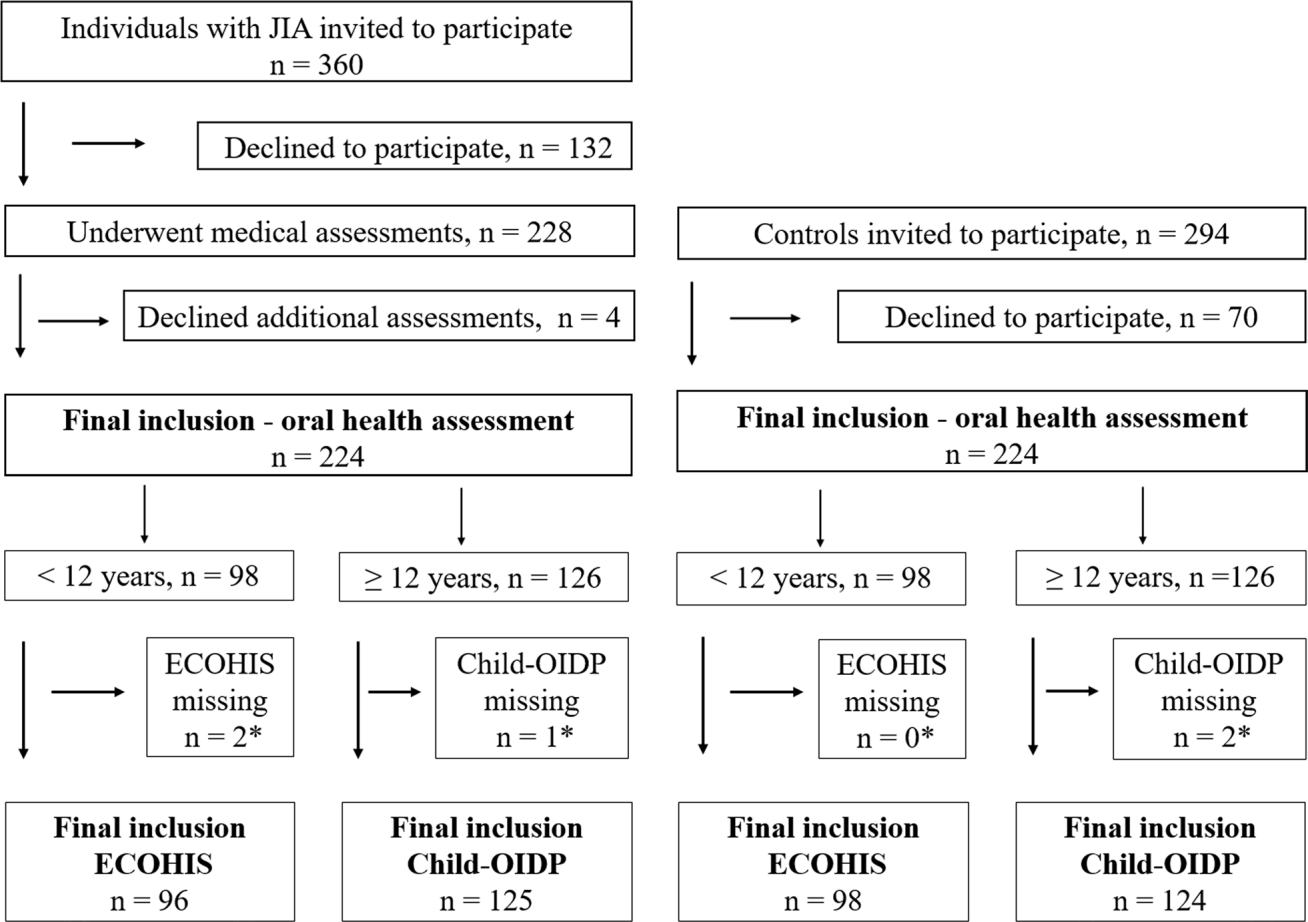
Participant flow diagram of children and adolescents with juvenile idiopathic arthritis (JIA) and controls. *Oral health related quality of life (OHRQoL) inventories having two or more unanswered items were excluded from the analysis. ECOHIS = Early Childhood Oral Health Impact Scale, Child-OIDP = Child Oral Impacts on Daily Performance

As depicted in Fig. 1, 228 individuals with JIA were submitted to the medical examination, resulting in a response rate of 63.3% (228/360) [30]. Concerning potential non-response bias, the mean age of the 132 eligible individuals with JIA who declined participation was 10.5 (SD = 3.5) years (*p* < 0.001). The proportion of females was slightly lower amongst the individuals with JIA who declined participation, compared to the participants with JIA (58.3% vs 59.2%, *p* = 0.027) [30].

Four participants with JIA did not undergo the oral examination, hence 224 participants were matched to controls (Fig. 1). The controls’ response rate was 76.2% (224/294) [30]. The matching resulted in 133 (59.4%) females in the JIA group and 134 (59.8%) females in the control group. The mean age for both groups was 12.0 years (both SD = 3.2) (*p* = 0.974) [30]. According to mother’s background of origin, 94.2% (211/224) of the pairs were matched [30]. As depicted in Fig. 1, 96 individuals with JIA and 98 controls were evaluated according to ECOHIS and 125 individuals with JIA and 124 controls were evaluated according to Child-OIDP.

Table 1 depicts the distribution of socio-demographic-, behavioral and oral health-related characteristics according to group affiliation for all participants aged 4–16 years. Higher proportions of mothers in the control group had high educational level compared to mothers of participants with JIA (73.8% versus 64.3%, *p* = 0.036). Corresponding figures among fathers were 57.1% *vs.* 42.5% (*p* = 0.003). Higher proportions of individuals with than without JIA reported gingival bleeding during toothbrushing (56.9% *vs.* 46.6%, *p* = 0.033). Concomitant diagnoses and medication use among the participants that may constitute an oral health threat are presented in a recently published article on dental caries in this study population [30].

**Table 1:** Distribution of socio-behavioral-, subjective clinical- and oral characteristics among individuals with juvenile idiopathic arthritis (JIA) and controls

Variable	JIA (n = 221)	Controls (n = 222)	*p*-value
*Educational level of caregivers, n (%)*			
Mother			
High school/vocational school	75 (35.7)	54 (26.2)	0.036^a^
University/college	135 (64.3)	152 (73.8)	
Father			
High school/vocational school	119 (57.5)	87 (42.9)	0.003^a^
University/college	88 (42.5)	116 (57.1)	
*Share household with, n (%)*			
Two caregivers in the household^b^	170 (79.4)	185 (84.1)	0.209
Only one caregiver in the household	44 (20.6)	35 (15.9)	
*Frequency of toothbrushing, n (%)*			
Twice a day, or more	163 (76.2)	170 (77.6)	0.719
Once a day or less/do not know	51 (23.8)	49 (22.4)	
*Frequency of tooth flossing during the last 3 months, n (%)*			
Daily or more	19 (8.9)	18 (8.3)	0.806
Several times weekly or less/do not know	194 (91.1)	200 (91.7)	
*During toothbrushing, gingival bleeding occurs, n (%)*			
Sometimes or more/do not know	120 (56.9)	102 (46.6)	0.033^a^
No	91 (43.1)	117 (53.4)	
*During toothbrushing, pain or discomfort occurs, n (%)*			
Yes/do not know	25 (11.9)	21 (9.6)	0.449
No	186 (88.2)	198 (90.4)	
*Dental caries*			
d_1-5_ft/D_1-5_FT^c^ = 0	118 (54.9)	124 (55.9)	0.838
d_1-5_ft/D_1-5_FT^c^ > 0	97 (45.1)	98 (44.1)	

^a^*p* < 0.05; χ^2^ test. ^b^Also includes living across two households, given two caregivers in both households. ^c^Decayed and/or filled teeth in the primary or permanent dentition, enamel caries included. Some variables had missing information

### Calibration

Four caries calibration exercises (described in Additional file 2) resulted in weighted Cohen’s kappa values of 0.61, 0.61, 0.91, and 0.65, respectively.

### Distribution of OHRQoL according to group affiliation

Table 2 depicts the distribution of the single items of ECOHIS and the total ECOHIS scores among the 4–11-year-olds according to group affiliation. Some single items (n = 6) differed significantly between the JIA group and controls with higher proportions of individuals being affected in the JIA group compared to the controls. The family impact score > 0 was also more frequently reported among participants with JIA (45.8% *vs.* 25.5%, *p* < 0.05). Internal consistency reliability of the ECOHIS total score in terms of Cronbach’s alpha was 0.87 in participants with JIA and 0.79 in the control group (Table 2).

**Table 2:** Distribution of the dichotomized items of the Early Childhood Oral Health Impact Scale (ECOHIS) and ECOHIS scores among individuals with juvenile idiopathic arthritis (JIA) (n = 96) and the controls (n = 98)

Item > 0*	JIA n (%)	Controls n (%)
*Child impact section*		
Pain in the teeth, mouth, or jaws?	59 (62.8)	49 (50.0)
Because of dental problems or the need for dental treatment		
Difficulty drinking hot or cold beverages?	26 (27.1)	18 (18.4)
Difficulty eating some foods?	32 (33.3)^a^	17 (17.4)
Difficulty pronouncing any words?	14 (14.6)^a^	5 (5.1)
Missed daycare, pre-school, or school?	25 (26.0)	17 (17.4)
Trouble sleeping?	18 (18.8)	17 (17.4)
Irritable or frustrated?	30 (31.6)	25 (25.5)
Avoided smiling or laughing when around other children?	13 (13.7)^a^	5 (5.1)
Avoided talking to other children?	8 (8.3)^a^	2 (2.0)
*Family impact section*		
Have you or another family member, due to dental problems or dental treatment of your child		
Been upset?	21 (21.9)	12 (12.2)
Felt guilty?	17 (17.7)	9 (9.2)
Taken time-off from work?	38 (39.6)^a^	19 (19.4)
Had financial impact on your family?	8 (8.33)^a^	1 (1.0)
*Child impact score > 0*	74 (77.1)	64 (65.3)
Cronbach’s alpha Child impact score	0.81	0.73
*Family impact score > 0*	44 (45.8)^a^	25 (25.5)
Cronbach’s alpha Family impact score	0.71	0.65
*ECOHIS total score > 0*	77 (80.2)	67 (68.4)
Cronbach’s alpha ECOHIS total score	0.87	0.79

^a^*p* < 0.05; χ^2^ test (no results were statistically significant). *The items are dichotomized as 0 not affected and 1 affected. Four participants had missing or replied “I don’t know” to one item in total

As shown in Table 3, neither the Child-OIDP SC nor the single item scores differed significantly between adolescents 12–16-years old with and without JIA, although a pattern towards more frequent impacts was observed in the JIA group compared to the controls. Cronbach’s alpha values of the Child-OIDP SC score were 0.83 in the participants with JIA and 0.79 in the control group (Table 3).

**Table 3:** Distribution of the dichotomized items of Child Oral Impacts on Daily Performance (Child-OIDP) and the simple count Child-OIDP score among individuals with juvenile idiopathic arthritis (n = 125) and the controls (n = 124)

Item > 0*	JIA n (%)	Controls n (%)
Eating	24 **(**19.2)	13 (10.5)
Speaking	7 (5.6)	5 (4.0)
Toothbrushing	14 (11.2)	11 (8.9)
Smiling and laughing	9 (7.2)	8 (6.5)
Sleeping and relaxing	11 (8.8)	4 (3.2)
Emotional balance	13 (10.4)	5 (4.0)
Socialization and contact with people	3 (2.4)	2 (1.6)
Study	6 (4.8)	2 (1.6)
*Child-OIDP simple count (SC) score > 0*	33 (26.4)	27 (21.8)
Cronbach’s alpha Child-OIDP SC score > 0	0.83	0.79

^a^*p* < 0.05; χ^2^ test. *The items are dichotomized as 0 not affected and 1 affected at least once or twice a month. None of the included participants had any items missing

### Discriminant validity of the OHRQoL measures

Additional file 8: Table S1 shows the frequency distribution of the ECOHIS- and Child-OIDP SC scores according to global measures of oral health, separately in children and adolescents with and without JIA. Among participants with JIA and controls, the ECOHIS scores were higher in parents who rated their child’s oral health as bad/reported dissatisfaction with appearance of teeth, compared to those who perceived their child’s oral health as good/ reported satisfaction with the appearance. The Child-OIDP SC scores were higher among participants reporting dissatisfaction with appearance of teeth, compared to participants reporting satisfaction with appearance of teeth.

### OHRQoL by group affiliation adjusted for socio-behavioral and clinical variables

Table 4 shows the results from unadjusted and adjusted ordinary logistic regression analyses of ECOHIS and Child-OIDP according to group affiliation. Increased odds ratios of having ECOHIS > 0 or OIDP > 0 were not statistically significant among participants with JIA in adjusted logistic regression analyses. Adjusted ordinary logistic regression analyses revealed a statistically significant association between d_1-5_ft/D_1-5_FT** > **0 and ECOHIS total score > 0 (OR = 3.39, 95% CI 1.40–8.22). Reporting pain or discomfort occurring during toothbrushing increased the likelihood of having Child-OIDP SC score > 0 (OR = 7.76, 95% CI 3.09–19.50). Adolescents with mothers reporting low educational level had significantly lower odds ratio for oral impacts according to Child-OIDP SC score (OR = 0.32, 95% CI 0.13–0.82). Corresponding to Table 4, negative binomial regression revealed almost similar results (Additional file 9: Table S1). However, adjusted negative binomial regressions showed a statistically significant increased incidence rate ratio of negatively impacted ECOHIS among participants with JIA compared to controls (IRR = 1.61, 95% CI 1.16–2.23) (Additional file 9).

**Table 4:** Group affiliation, socio-behavioral, and clinical characteristics in relation to the outcome variable Early Childhood Oral Health Impact Scale (ECOHIS) total score > 0 and Child Oral Impacts on Daily Performance (Child-OIDP) simple count (SC) score > 0

	Unadjusted regression						Adjusted regression					
	ECOHIS total score > 0			Child-OIDP SC score > 0			ECOHIS total score > 0^a^			Child-OIDP SC score > 0^b^		
	OR	95% CI	*p*-value	OR	95% CI	*p*-value	OR	95% CI	*p*-value	OR	95% CI	*p*-value
*Group affiliation*												
Control group	Ref			Ref			Ref			Ref		
JIA	1.88	(0.97–3.64)	0.064	1.29	(0.72–2.32)	0.396	1.95	(0.94–4.04)	0.072	0.99	(0.46–2.17)	0.987
*Educational level of mother*												
University/college	Ref			Ref			Ref			Ref		
High school/vocational school	1.29	(0.64–2.63)	0.477	0.63	(0.30–1.30)	0.209	0.87	(0.39–1.92)	0.729	0.32	(0.13–0.82)	**0.018**
*Educational level of father*												
University/college	Ref			Ref			Ref			Ref		
High school/vocational school	1.70	(0.89–3.27)	0.111	1.20	(0.63–2.26)	0.582	1.44	(0.64–3.25)	0.377	1.71	(0.76–3.83)	0.194
*Household structure*^*c*^												
Two caregivers^c^	Ref			Ref								
One caregiver	1.75	(0.55–5.59)	0.347	0.98	(0.45–2.14)	0.963						
*Frequency of toothbrushing, n (%)*												
Twice a day, or more	Ref			Ref			Ref					
Once a day or less/do not know	2.45	(1.07–5.61)	**0.035**	1.10	(0.55–2.23)	0.785	2.54	(0.99–6.54)	0.054			
*Frequency of tooth flossing during the last 3 months, n (%)*												
Daily or more	Ref			Ref								
Several times weekly or less/do not know	0.81	(0.17–3.90)	0.792	0.82	(0.34–1.97)	0.654						
*During toothbrushing, gingival bleeding occurs*												
Never	Ref			Ref			Ref			Ref		
Sometimes or more/do not know	1.38	(0.69–2.75)	0.363	1.66	(0.83–3.31)	0.149	1.10	(0.52–2.34)	0.810	1.38	(0.61–3.12)	0.447
*During toothbrushing, pain or discomfort occurs*												
No	Ref			Ref						Ref		
Yes/do not know	1.24	(0.24–6.38)	0.801	9.69	(4.25–22.10)	** < 0.001**				7.76	(3.09–19.50)	** < 0.001**
*Dental caries*												
d_1-5_ft/D_1-5_FT^d^ = 0	Ref			Ref			Ref			Ref		
d_1-5_ft/D_1-5_FT^d^ > 0	3.43	(1.44–8.19)	**0.006**	1.96	(1.05–3.67)	**0.036**	3.39	(1.40–8.22)	**0.007**	1.70	(0.81–3.56)	0.163

Unadjusted and adjusted ordinary logistic regressions

^a^ECOHIS > 0 regressed on group affiliation and adjusted for socio-behavioral factors (parental educational level, frequency of toothbrushing and bleeding during toothbrushing) and dental caries. McFadden’s R^2^ = 8.9%. ^b^OIDP > 0 regressed on group affiliation and adjusted for socio-behavioral factors (parental educational level, bleeding during toothbrushing and pain or discomfort during toothbrushing) and dental caries. McFadden’s R^2^ = 14.1%. ^c^Also includes living across two households, given two caregivers in both households. ^d^Decayed and/or filled teeth in the primary or permanent dentition, enamel caries included. OR = odds ratios. CI = confidence interval. JIA = juvenile idiopathic arthritis. P-values < 0.05 are marked in bold

A significant two-way interaction between group affiliation and gender on Child-OIDP SC score was revealed by logistic regression (*p* = 0.015). Stratified analyses revealed that females had higher odds ratio for having Child-OIPD SC score > 0 compared to males, among participants with JIA (OR = 6.12, 95% CI 2.29–16.30, *p* < 0.001) (not presented in any table). Amongst the controls, females had higher odds ratio for having Child-OIPD SC > 0 compared to males (OR = 1.23, 95% CI 0.52–2.90), although not statistically significant (not presented in any table).

### Disease-specific features in relation to OHRQoL

Table 5 shows the results from adjusted ordinary logistic regression analyses of disease-specific features in relation to ECOHIS and Child-OIDP among children and adolescents with JIA. Covariates were age, gender and parental educational level, and the JIA-specific variables were adjusted separately. Children with ongoing or ever used biologic DMARDs (disease-modifying antirheumatic drugs) were more likely than their counterparts without ongoing or ever used biologic DMARDs to have ECOHIS > 0 (OR = 7.59, 95% CI 1.77–32.67, *p* = 0.006 and OR = 9.20, 95% CI 1.93–43.97, *p* = 0.005). Adolescents categorized ‘not oligoarthritis persistent’ (comprising oligoarthritis extended, polyarthritis RF positive and RF negative, psoriatic arthritis, and enthesitis-related arthritis) had statistically significantly increased odd ratio of having Child-OIDP SC > 0, compared to participants in the JIA category oligoarthrits persistent (OR = 6.29, 95% CI 1.83–21.63). Adolescents with continued activity or flare revealed statistically significantly increased odds ratio of having Child-OIDP SC > 0, compared to participants with inactive disease or remission (OR = 3.01, 95% CI 1.15–7.89). This also applied to the self-reported pain (VAS pain > 0), compared to no pain (VAS pain = 0), and self-reported physical disability (CHAQ > 0), compared to no disability (CHAQ = 0) (OR = 4.39, 95% CI 1.20–16.14, OR = 4.21, 95% CI 1.40–12.68, respectively). Corresponding to Table 5, negative binomial regression revealed almost similar results (Additional file 10: Table S1).

**Table 5:** JIA disease-specific features and dental caries in relation to Early Childhood Oral Health Impact Scale (ECOHIS) total score > 0 and Child Oral Impacts on Daily Performance (Child-OIDP) simple count (SC) score > 0

	ECOHIS total score > 0							Child-OIDP SC score > 0						
	Unadjusted regressions				Adjusted regressions^a^			Unadjusted regressions				Adjusted regressions^a^		
	n	OR	95% CI	*p*-value	OR	95% CI	*p*-value	n	OR	95% CI	*p*-value	OR	95% CI	*p*-value
*JIA category*														
Oligoarthritis persistent	32	Ref			Ref			44	Ref			Ref		
Not oligoarthritis persistent^b^	45*	3.64	(1.09–12.08)	**0.035**	3.32	(0.82–13.40)	0.092	62**	6.32	(1.99–20.01)	**0.002**	6.29	(1.83–21.63)	**0.004**
*Age at JIA onset*														
≤ 6 years	64	Ref			Ref			46	Ref			Ref		
> 6 years	32	0.82	(0.29–2.36)	0.719	0.61	(0.17–2.11)	0.430	79	1.03	(0.45–2.35)	0.952	1.52	(0.56–4.14)	0.414
*Disease duration*														
≤ 5 years	57	Ref			Ref			60	Ref			Ref		
> 5 years	39	3.13	(0.94–10.34)	0.062	2.81	(0.77–10.27)	0.117	65	0.97	(0.44–2.17)	0.948	0.87	(0.34–2.19)	0.762
*Steroids, ever used*														
No steroids ever used	74	Ref			Ref			99	Ref			Ref		
Steroids ever used	22	0.79	(0.25–2.53)	0.696	0.57	(0.15–2.14)	0.409	26	1.32	(0.51–3.41)	0.572	2.32	(0.79–6.88)	0.128
*DMARDs, ongoing*														
No sDMARDs nor bDMARDs ongoing	26	Ref			Ref			49	Ref			Ref		
sDMARDs, but no bDMARDs ongoing	34	2.04	(0.64–6.55)	0.230	2.00	(0.57–7.02)	0.280	27	2.68	(0.95–7.58)	0.063	1.38	(0.41–4.65)	0.601
bDMARDs ongoing^c^	36	5.82	(1.38–24.56)	**0.016**	7.59	(1.77–32.67)	**0.006**	49	1.27	(0.49–3.29)	0.630	1.20	(0.40–3.62)	0.743
*DMARDs, ever used*														
No sDMARDs nor bDMARDs ever used	15	Ref			Ref			37	Ref			Ref		
sDMARDs, but no bDMARDs ever used	43	2.52	(0.70–9.01)	0.155	3.41	(0.91–12.79)	0.069	38	2.50	(0.87–7.21)	0.090	1.20	(0.37–3.82)	0.764
bDMARDs ever used^c^	38	5.67	(1.30–24.66)	**0.021**	9.20	(1.93–43.97)	**0.005**	50	1.35	(0.47–3.88)	0.573	1.15	(0.35–3.72)	0.822
*Disease status*^*d*^														
Inactive disease/remission on/off medication	59	Ref			Ref			74	Ref			Ref		
Continued activity/flare	37	2.81	(0.85–9.32)	0.091	2.65	(0.81–8.65)	0.106	51	3.62	(1.57–8.34)	**0.003**	3.01	(1.15–7.89)	**0.025**
*MDgloVAS*														
VAS = 0	63	Ref			Ref			79	Ref			Ref		
VAS > 0	33	2.27	(0.68–7.54)	0.182	2.29	(0.71–7.44)	0.168	46	2.74	(1.21–6.23)	**0.016**	1.98	(0.77–5.08)	0.156
*VAS pain*														
VAS = 0	36	Ref			Ref			45	Ref			Ref		
VAS > 0	60	1.27	(0.46–3.56)	0.646	1.13	(0.38–3.42)	0.825	76	4.16	(1.46–11.86)	**0.008**	4.39	(1.20–16.14)	**0.026**
*PRgloVAS*														
VAS = 0	25	Ref			Ref			34	Ref			Ref		
VAS > 0	71	1.91	(0.65–5.61)	0.238	1.69	(0.52–5.50)	0.386	87	3.38	(1.08–10.58)	**0.037**	2.54	(0.65–9.97)	0.182
*CHAQ*^*e*^														
CHAQ = 0	37	Ref			Ref			54	Ref			Ref		
CHAQ > 0	59	1.58	(0.57–4.36)	0.382	1.76	(0.58–5.37)	0.322	71	6.38	(2.26–18.06)	** < 0.001**	4.21	(1.40–12.68)	**0.011**
*Dental caries*														
d_1-5_ft/D_1-5_FT^f^ = 0	65	Ref			Ref			54	Ref			Ref		
d_1-5_ft/D_1-5_FT^f^ > 0	29	2.60	(0.69–9.87)	0.160	1.97	(0.49–7.97)	0.342	68	2.04	(0.84–4.96)	0.117	1.63	(0.57–4.65)	0.362

Unadjusted and adjusted ordinary logistic regressions

^a^Adjusted for: gender, age and educational level of mother and educational level of father. For the adjusted models McFadden’s R^2^ varied between 7.0%-15.4% (ECOHIS total score > 0) and 10.6%-19.3% (Child-OIDP SC score > 0)*. *^b^Includes oligoarthritis extended, polyarthritis RF positive and RF negative, psoriatic arthritis, and enthesitis-related arthritis. ^c^With or without sDMARDs. ^d^Disease activity according to Wallace [66] and the American College of Rheumatology provisional criteria [67]. ^e^Self-reported physical disability measured with the disease-specific and validated Childhood Health Assessment Questionnaire (CHAQ) (0 = no difficulty and 3 = unable to do) [34]. ^f^Decayed and/or filled teeth in the primary or permanent dentition, enamel caries included. Some registrations are missing. *Oligoarthritis extended (n = 11), polyarthritis, RF positive (n = 1) and RF negative (n = 24), psoriatic arthritis (n = 3), and enthesitis-related arthritis (n = 6). **Oligoarthritis extended (n = 11), polyarthritis, RF positive (n = 1) and RF negative (n = 26), psoriatic arthritis (n = 5), and enthesitis-related arthritis (n = 17). RF = rheumatoid factor. sDMARDs = synthetic disease-modifying antirheumatic drugs. bDMARDs = biologic disease-modifying antirheumatic drugs. MDgloVAS = physician's global assessment of disease activity visual analogue scale (VAS). VAS pain = patient/parent-reported pain intensity. PRgloVAS = patient's global assessment of overall wellbeing. All visual analogue scales were measured on a 21-numbered circle VAS (0–10). CHAQ = Childhood Health Assessment Questionnaire (0 = no disability, 3 = maximum disability). OR = odds ratios. CI = confidence interval. JIA = juvenile idiopathic arthritis. P-values < 0.05 are marked in bold

## Discussion

To our knowledge, this is one of the first studies to estimate the proportion and socio-, behavioral and clinical covariates of OHRQoL in young people with and without JIA. Present findings based on multiple variable logistic regression analysis did not confirm the hypothesis that children and adolescents with JIA have poorer OHRQoL than their counterparts without JIA. According to the ECOHIS scores this conclusion is unsure, since the adjusted binominal regression analyses showed an increased risk of impaired ECOHIS scores amongst the 4–11-year-olds with JIA compared to controls. Although the proportions who confirmed impacts according to ECOHIS and Child- OIDP scores were substantial across both groups, and always tended to be higher among children and adolescents with than without JIA, neither scale scores varied by group affiliation in the adjusted logistic regression analyses. Independent of JIA status, the likelihood of impaired OHRQoL increased by caries experience among the younger participants. Among the older participants, Child OIDP associated negatively and positively with maternal education and having pain/discomfort during toothbrushing, respectively. Although the socio-behavioral and clinical distribution of OHRQoL scores were less variant across the two groups of participants, female adolescents with JIA were more likely than males to report oral impacts according to Child OIDP. The corresponding association in the control group was not significant. Specifically, for adolescents with JIA, continued activity or flare was found to adversely affect Child-OIDP, indicating that sub-groups of JIA may have reduced OHRQoL, Also, self-reported outcome measures of the disease (VAS pain and CHAQ) associated with Child-OIDP.

Important strengths of the study were that the study group of individuals with JIA was relatively large and might be representative of the Norwegian population of children and adolescents with JIA. Also, a well-matched control group and the adjustment of various socio-demographic, behavioral, and clinical covariates strengthened the results [27, 36]. The present study revealed that both ECOHIS and Child-OIDP discriminated significantly according to global measures of oral health indicating satisfactory psychometrical properties of both instruments across the investigated groups. Other strengths were the meticulous calibration of caries examiners before and during the study and the use of the previous validated OHRQoL instruments in the context of Norwegian children and adolescents, which also showed a satisfactory internal consistency reliability [19]. Among limitations to be considered were a potential non-response bias among the participants with JIA [37] and the small subgroups of JIA disease categories that might have prevented valid interpretation of the relation between various disease categories and OHRQoL. Furthermore, considering the multilevel influences of oral health, other potential confounding variables of OHRQoL have not been adjusted for in the present study [38]. As a sub-study, the sample size calculation in the present article was based in caries figures and not on OHRQoL. This questions the study's statistical power and needs to be kept in mind while interpreting the results. Finally, evaluation of OHRQoL among the youngest study participants were only conducted by parental proxy-reporting and may reduce the quality of data collected. Thus, evidence suggests that parents tend to underestimate the impact of children’s oral problems as their perspective is different and they might have limited knowledge of their children’s social and emotional well-being [39].

Some descriptive studies have been conducted in European countries evaluating OHRQoL by the application of ECOHIS [19, 40–43]. Except for one study conducted in Norway by Skeie et al. [19], all of these studies evaluated OHRQoL in preschool children below the age of 6 years, hence complicating direct comparisons with the present study. Whereas the proportion of ECOHIS child impacts in this study amounted to 77.1% in the JIA group and 65.3% among controls, the corresponding figure among children in the study by Skeie et al. [19] was 71.0%. In contrast, the proportions of adolescents with and without JIA having impacts according to the Child-OIDP in the present study were 26.4% and 21.8%, a higher rate of 42.7% was found among adolescents in the study by Skeie et al. [19]. These differences in children’s and adolescents’ impact proportions might be attributed to minor age differences in the study groups investigated (also 17- and 18-year-olds were included in the study by Skeie et al.). The subjective and dynamic aspects of OHRQoL is based on individual experiences values and perceptions [44]. Thus, OHRQoL varies across groups within and across countries, as well as over time [44]. Nevertheless, many studies using Child-OIDP have been published [45–47]. A recent systematic review on OHRQoL in adolescents measured by use of Child-OIDP worldwide, reported prevalence rates of impacts within a wide range among adolescents 12 years and older, 15.8%-87% respectively [47].

Few studies have compared sub-scale OHRQoL scores with healthy controls. As shown in Table 2 physical- and psychosocial aspects of the Child impact scores and ‘taken time off from work’ from the family impact sub-scale were the most frequently reported impacts in both groups of younger children investigated. However, the prevalence of child impacts were consistently higher in the JIA group than among the controls, particularly regarding impacts related to physical and psycho-social functioning. Also, according to Table 3, the number of adolescents reporting impacts on the single OIDP items tended to be higher among participants with JIA, compared to controls. Physical aspects in terms of difficulty eating was most frequent among the 8 single OIDP scores. Impact of the function “eating” has also been demonstrated to be related to TMJ arthritis [23]. Although neither scale scores varied by group affiliation in the adjusted logistic regression analyses, children and adolescents with JIA seems to carry a particular burden regarding physical and psycho-social functioning. This is consistent with previous evidence that rheumatic diseases may result in important functional and psycho-social impairments, though examined among adult populations [48].

The present results showed that neither the ECOHIS- nor the Child OIDP scores varied by group affiliation in adjusted logistic regression analyses. This supports the findings of Santos et al. [22], who observed no significant difference in OHRQoL scores between individuals with JIA and controls, as perceived by their caregivers. However, comparisons with other studies are problematic as various OHRQoL instruments have been utilized, adjustment for covariates is seldom implemented and participants in the relevant studies are categorized specifically according to oral health status (e.g., JIA + TMJ arthritis, JIA + periodontitis) [22–25]. A plausible contributing factor of comparable OHRQoL between participants with JIA and controls is improved therapeutic effect, especially increased efficacy of pharmaceutical drugs, in the management of JIA [49, 50].

Independent of group affiliation, caries experience associated significantly with impaired OHRQOL amongst 4–11-year-olds in the present study. This corresponds with previous studies conducted across cultural contexts [51, 52]. The ECOHIS scale was originally developed to assess the impact of dental caries but have been widely used as generic OHRQoL instrument [45]. A systematic review by Kumar et al. [36] found that higher parental education associated with better OHRQoL in children. Contrary, we found that adolescents having lower educated mothers were less likely than their counterparts with higher educated mothers to report oral impacts according to Child OIDP. However, research findings in this area are conflicting and some studies have documented insignificant associations between parental socio-economic status and children’s OHRQoL [36, 51].

Female adolescents with JIA were significantly more likely than male adolescents to report oral impacts according to Child-OIDP; the corresponding association in the control group was nonsignificant. Other studies reporting poorer OHRQoL among female participants compared to males, by the employment of Child-OIDP, consider females more sensitive to problems and appearance than males [53, 54]. Even in the biological era, pain and depressive symptoms, known to impact the quality of life, are common in JIA patients [55–57]. Comparable to findings in the general pediatric population, research focusing on young individuals with JIA indicates, although inconsistently, a gender difference; females report such complaints more frequently than males, and the complaints become apparent in their adolescent years [58–63]. This may provide an explanation for female adolescents with JIA reporting poorer OHRQoL in this study.

Several JIA-specific covariates related to disease activity and patient/parent-reported pain, and functional disability were associated with Child-OIDP in the present study. OHRQoL is recognized to be part of health-related quality of life [64, 65], hence the association between patient/parent-reported covariates and OHRQoL was anticipated. In the present study biologic DMARDs, ongoing or ever used, were shown to be associated with impaired ECOHIS scores amongst the younger population with JIA. Thus, a more severe disease course as indicated by biologic DMARDs ongoing or ever used, is suggested to be associated with OHRQoL. No disease-specific inventory exists to evaluate OHRQoL in individuals with JIA. Accordingly, various impacts of JIA-specific features on OHRQoL are not necessarily identified by the generic instruments utilized in this study.

## Conclusions

This study did not provide consistent evidence to confirm the hypothesis that children and adolescents with JIA are more likely to have impaired OHRQoL compared to their peers without JIA. However, female adolescents with JIA were more likely than males to report impacts on OHRQoL. Furthermore, within the JIA group, adolescents with continued disease activity, flare or reporting pain, or physical disability, had higher risk than their counterparts of impaired OHRQoL.

## Supplementary Information

**Additional file 1.** Sample size calculation.

**Additional file 2.** Calibration.

**Additional file 3.** **Table S1**. Categories for socio-behavioral characteristics, as originally coded and as re-coded for analyses.

**Additional file 4.** **Table S1**. Categories for Early Childhood Oral Health Impact Scale (ECOHIS) (4–11 years) and questions regarding satisfaction with oral health (global measures), as originally coded and as re-coded for analyses.

**Additional file 5.** **Table S1**. Categories for Child Oral Impacts on Daily Performances (Child-OIDP) (12–16 years) and questions regarding satisfaction with oral health (global measures), as originally coded and as re-coded for analyses.

**Additional file 6.** Description of JIA-specific background variables.

**Additional file 7.** **Table S1**. Categories for JIA-specific background variables, as originally coded and re-coded for analyses.

**Additional file 8.** **Table S1**. Discriminant validity of the Early Childhood Oral Health Impact Scale (ECOHIS) and Child Oral Impacts on Daily Performances (Child-OIDP) according to the global measures and group affiliation.

**Additional file 9.** **Table S1**. Group affiliation, socio-behavioral, and clinical characteristics in relation to the outcome variable Early Childhood Oral Health Impact Scale (ECOHIS) total score and Child Oral Impacts on Daily Performance (Child-OIDP) simple count (SC) score. Unadjusted and adjusted negative binominal regressions.

**Additional file 10.** **Table S1**. Disease-specific features and dental caries in relation to the outcome variable Early Childhood Oral Health Impact Scale (ECOHIS) total score and Child Oral Impacts on Daily Performance (Child-OIDP) simple count (SC) score. Unadjusted and adjusted negative binominal regressions.

## Data Availability

The datasets used and/or analyzed during the current study are available from the corresponding author on reasonable request.

## Abbreviations

bDMARDs: Biologic disease-modifying antirheumatic drugs
CHAQ: Childhood Health Assessment Questionnaire
Child-OIDP: Child Oral Impacts on Daily Performances
COHQoL: Child Oral Health-related Quality of Life measure
CPQ_11-14_: Child Perception Questionnaire
DMARDs: Disease-modifying antirheumatic drugs
BW: Bitewing radiographs
CHAQ: Childhood Health Assessment Questionnaire
CI: Confidence interval
dfs/DFS: Decayed and filled surfaces
ECOHIS: Early Childhood Oral Health Impact Scale
ILAR: International League of Associations for Rheumatology
IRR: Incidence rate ratios
JIA: Juvenile idiopathic arthritis
MDgloVAS: Physician’s global assessment of disease activity visual analogue scale
NorJIA: The Norwegian JIA Study (Temporo-mandibular Involvement, Oral Health, Uveitis, Bone Health and Quality of Life in Children with Juvenile Idiopathic Arthritis)
OHRQoL: Oral health-related quality of life
OR: Odds ratios
PRgloVAS: Patient/parent-reported global assessment of overall wellbeing visual analogue scale
RF: Rheumatoid Factor
SC: Simple count
SD: Standard deviations
sDMARDs: Synthetic disease-modifying antirheumatic drugs
TMD: Temporomandibular disorder
TMJ: Temporomandibular joint
VAS: Visual analogue scale
VAS pain: Patient/parent-reported pain intensity visual analogue scale

## Footnotes

1 The Norwegian JIA Study – Temporo-mandibular Involvement, Oral Health, Uveitis, Bone Health and Quality of Life in Children with Juvenile Idiopathic Arthritis (JIA).
