# Supplementary material for: Oral health-related quality of life in 4–16-year-olds with and without juvenile idiopathic arthritis
Source: BMC Oral Health. 2022 Sep 6;22:387. doi: 10.1186/s12903-022-02400-1 (PMC9450232; doi:10.1186/s12903-022-02400-1)
Supplement: Supplementary file 1 — Additional file 1. Sample size calculation. [file 12903_2022_2400_MOESM1_ESM.docx]

**Additional file 1**

*Sample size calculation*

The sample size calculation was based on caries figures in children and adolescents with JIA. For primary dentition, the estimate was based on a study by Welbury et al. (1), reporting a mean dmft (decayed at dentin level) of 1.46 in the JIA group and 0.56 in controls with corresponding standard deviations (SD) of 2.58 and 0.96. The significance level and the statistical power were decided on 5% and 80%, respectively. The proposed sample size was 75 participants in each group, applying a two-sided student sample t-test. Sample size calculation of the permanent dentition was based on a study by Leksell et al. (2) reporting caries prevalence (decayed at dentin level) in the first permanent molars of 49% in the JIA group and 27% in the control group. Assumptions of alpha of 5% and power of 80% resulted in an estimated sample size of 76 for each group by applying a two-sample comparison of percentages. Altogether, for both the primary and the permanent dentition, the suggested number of participants was 182 in each group (JIA/controls) in anticipation of a dropout rate of 20% (missing/canceled appointments).

1. Welbury RR, Thomason JM, Fitzgerald JL, Steen IN, Marshall NJ, Foster HE. Increased prevalence of dental caries and poor oral hygiene in juvenile idiopathic arthritis. Rheumatology (Oxford). 2003;42(12):1445-51.

2. Leksell E, Ernberg M, Magnusson B, Hedenberg-Magnusson B. Intraoral condition in children with juvenile idiopathic arthritis compared to controls. Int J Paediatr Dent. 2008;18(6):423-33.
