# Supplementary material for: Oral health-related quality of life in 4–16-year-olds with and without juvenile idiopathic arthritis
Source: BMC Oral Health. 2022 Sep 6;22:387. doi: 10.1186/s12903-022-02400-1 (PMC9450232; doi:10.1186/s12903-022-02400-1)
Supplement: Supplementary file 2 — Additional file 2. Calibration. [file 12903_2022_2400_MOESM2_ESM.docx]

**Additional file 2**

*Calibration*

A plastic-coated instruction sheet of the written description of the five-grade caries diagnosis system with associated photographs (1) was delivered to all examiners. In the initial theoretical session, gradings of available bitewing radiographs and clinical pictures of tooth surfaces were discussed, and an expert gave feedback.

The examiners then evaluated radiographs and pictures of caries and compared their findings with "the expert reference". Before and during the study period, in total, four sessions of caries calibration exercises were performed (Test Caries 1, 2, 3, 4). Most data for the caries reliability assessment were based on 71 schoolchildren presenting both primary and permanent teeth. Also, bitewing radiographs (n = 21) of primary and permanent teeth and surfaces of extracted teeth (n = 9) were included. For Test Caries 1, only one examiner was included, and this examiner was compared to "an expert reference". Test Caries 2 was based on the inter-examiner examiner evaluation. Test Caries 3 was an intra-examiner assessment of each examiner with an interval between, while Test Caries 4 was based on a comparison to "an expert reference".

1. Amarante E, Raadal M, Espelid I. Impact of diagnostic criteria on the prevalence of dental caries in Norwegian children aged 5, 12 and 18 years. Community Dent Oral Epidemiol. 1998;26(2):87-94.
