# Supplementary material for: Oral health-related quality of life in 4–16-year-olds with and without juvenile idiopathic arthritis
Source: BMC Oral Health. 2022 Sep 6;22:387. doi: 10.1186/s12903-022-02400-1 (PMC9450232; doi:10.1186/s12903-022-02400-1)
Supplement: Supplementary file 3 — Additional file 3. Table S1. Categories for socio-behavioral characteristics, as originally coded and as re-coded for analyses. [file 12903_2022_2400_MOESM3_ESM.docx]

**Additional file 3**

Table S1. Categories for socio-behavioral characteristics, as originally coded and as re-coded for analyses.

| Variables | Categories | Original code | New code |
| --- | --- | --- | --- |
| Educational level of mother/father | Primary school | 1 | 1 |
|  | High school/vocational school | 2 | 1 |
|  | University/college (≥4years) | 3 | 0 |
|  | University/college (≤5 years) | 4 | 0 |
|  | Unknown | 90 | Missing |
|  | Missing | 99 | Missing |
| Share household with^*^ | Mother and father | 1 | 0 |
|  | Only mother | 2 | 1 |
|  | Only father | 3 | 1 |
|  | Other, specify | 4 | 0/1^*^ |
| Frequency of toothbrushing | Never | 1 | 1 |
|  | Most days | 2 | 1 |
|  | Once a day | 3 | 1 |
|  | Twice a day, or more | 4 | 0 |
|  | Do not know | 90 | 1 |
|  | Missing | 99 | Missing |
| Frequency of tooth flossing during the last 3 months | Several times daily | 1 | 0 |
|  | Twice a day | 2 | 0 |
|  | Daily | 3 | 1 |
|  | Several times weekly | 4 | 1 |
|  | Several times a month, but not weekly | 5 | 1 |
|  | Seldom | 6 | 1 |
|  | Never | 7 | 1 |
|  | Do not know | 90 | 1 |
|  | Missing | 99 | Missing |
| During toothbrushing, gingival bleeding occurs | Every day | 1 | 1 |
|  | Most days | 2 | 1 |
|  | Once a week | 3 | 1 |
|  | Sometimes | 4 | 1 |
|  | Never | 5 | 0 |
|  | Do not know | 90 | 1 |
|  | Missing | 99 | Missing |
| The two questions below are merged and form the basis of “During toothbrushing, pain or discomfort occurs” | | | |
| Question given to the participants ≥ 12 years: **“**Do you sometimes experience pain or discomfort during toothbrushing?” | Yes | 1 | 1 |
|  | No | 2 | 0 |
|  | Do not know | 90 | 1 |
|  | Missing | 99 | Missing |
| Question given to the participants < 12 years: “Impression of child’s experience during toothbrushing?” | Painful | 1 | 1 |
|  | Unpleasant | 2 | 1 |
|  | Okay | 3 | 0 |
|  | Do not know | 90 | 1 |
|  | Missing | 99 | Missing |
| Additional questions regarding satisfaction with oral health (global measures) | | | |
|  |  |  |  |
| How do you consider your/your child’s oral health? | Very good | 1 | 0 |
|  | Good | 2 | 0 |
|  | Not good or bad | 3 | 1 |
|  | Bad | 4 | 1 |
|  | Very bad | 5 | 1 |
|  | Missing | 99 | Missing |
|  |  |  |  |
| How satisfied or dissatisfied are you with the appearance of your/your child’s teeth? | Very satisfied | 1 | 0 |
|  | Satisfied | 2 | 0 |
|  | Not satisfied or dissatisfied | 3 | 1 |
|  | Dissatisfied | 4 | 1 |
|  | Very Dissatisfied | 5 | 1 |
|  | Missing | 99 | Missing |

**The variable “Share household with” was transformed into “Two caregivers in the household” (0), which also includes living across two households with two caregivers in each households, or ”Only one caregiver in the household” (1). The answer “Other, specify” was evaluated and recoded accordingly.*
