# Supplementary material for: Oral health-related quality of life in 4–16-year-olds with and without juvenile idiopathic arthritis
Source: BMC Oral Health. 2022 Sep 6;22:387. doi: 10.1186/s12903-022-02400-1 (PMC9450232; doi:10.1186/s12903-022-02400-1)
Supplement: Supplementary file 4 — Additional file 4. Table S1. Categories for Early Childhood Oral Health Impact Scale (ECOHIS) (4–11 years) and questions regarding satisfaction with oral health (global measures), as originally coded and as re-coded for analyses. [file 12903_2022_2400_MOESM4_ESM.docx]

**Additional file 4**

Table S1. Categories for Early Childhood Oral Health Impact Scale (ECOHIS) (4–11 years) and questions regarding satisfaction with oral health (global measures), as originally coded and as re-coded for analyses.

| Variables | | Categories | Original code | New code |
| --- | --- | --- | --- | --- |
|  |  |  |  |  |
| **Child impact section** | | | | |
| 1. How often has your child had pain in the teeth, mouth, or jaws | | Never | 1 | 0 |
|  |  | Hardly ever | 2 | 1 |
|  |  | Occasionally | 3 | 1 |
|  |  | Often | 4 | 1 |
|  |  | Very often | 5 | 1 |
|  |  | Don’t know | 90 | Missing |
|  |  | Missing | 99 | Missing |
|  | |  |  |  |
| How often has your child ………because of dental problems or dental treatments | 2. had difficulty drinking hot or cold beverages  3. had difficulty eating some foods  4. had difficulty pronouncing any words  5. missed daycare, preschool, or school | Never | 1 | 0 |
|  |  | Hardly ever | 2 | 1 |
|  |  | Occasionally | 3 | 1 |
|  |  | Often | 4 | 1 |
|  |  | Very often | 5 | 1 |
|  |  | Don’t know | 90 | Missing |
|  |  | Missing | 99 | Missing |
|  |  |  |  |  |
|  |  |  |  |  |
| How often has your child ………because of dental problems or dental treatments | 6. had trouble sleeping  7. been irritable or frustrated | Never | 1 | 0 |
|  |  | Hardly ever | 2 | 1 |
|  |  | Occasionally | 3 | 1 |
|  |  | Often | 4 | 1 |
|  |  | Very often | 5 | 1 |
|  |  | Don’t know | 90 | Missing |
|  |  | Missing | 99 | Missing |
|  |  |  |  |  |
| How often has your child ………because of dental problems or dental treatments | 8. avoided smiling or laughing when around other children  9. avoided talking with other children | Never | 1 | 0 |
|  |  | Hardly ever | 2 | 1 |
|  |  | Occasionally | 3 | 1 |
|  |  | Often | 4 | 1 |
|  |  | Very often | 5 | 1 |
|  |  | Don’t know | 90 | Missing |
|  |  | Missing | 99 | Missing |
|  |  |  |  |  |
| **Parent impact section** | | | | |
| How often have you or another family member......because of your child's dental problems or dental treatments? | 10. been upset?  11. felt guilty? | Never | 1 | 0 |
|  |  | Hardly ever | 2 | 1 |
|  |  | Occasionally | 3 | 1 |
|  |  | Often | 4 | 1 |
|  |  | Very often | 5 | 1 |
|  |  | Don’t know | 90 | Missing |
|  |  | Missing | 99 | Missing |
|  |  |  |  |  |
| How often .... | 12. have you or another family member taken time-off from work because of your child's dental problems or dental treatments  13. has your child had dental problems or dental treatments that had a financial impact on your family? | Never | 1 | 0 |
|  |  | Hardly ever | 2 | 1 |
|  |  | Occasionally | 3 | 1 |
|  |  | Often | 4 | 1 |
|  |  | Very often | 5 | 1 |
|  |  | Don’t know | 90 | Missing |
|  |  | Missing | 99 | Missing |
