# Supplementary material for: Oral health-related quality of life in 4–16-year-olds with and without juvenile idiopathic arthritis
Source: BMC Oral Health. 2022 Sep 6;22:387. doi: 10.1186/s12903-022-02400-1 (PMC9450232; doi:10.1186/s12903-022-02400-1)
Supplement: Supplementary file 5 — Additional file 5. Table S1. Categories for Child Oral Impacts on Daily Performances (Child-OIDP) (12–16 years) and questions regarding satisfaction with oral health (global measures), as originally coded and as re-coded for analyses. [file 12903_2022_2400_MOESM5_ESM.docx]

**Additional file 5**

Table S1. Categories for Child Oral Impacts on Daily Performances (Child-OIDP) (12–16 years) and questions regarding satisfaction with oral health (global measures), as originally coded and as re-coded for analyses.

| Variables | Categories | Original code | New code |
| --- | --- | --- | --- |
|  |  |  |  |
| **“During the past 3 months, how often have problems with your mouth or teeth caused you any difficulty with: ….”** | | | |
|  | | | |
| 1. eating | Never | 0 | 0 |
|  | Once or twice a month | 1 | 1 |
|  | Once or twice a week | 2 | 1 |
|  | Every day/almost every day | 3 | 1 |
|  | Missing | 99 | Missing |
|  |  |  |  |
| 2. speaking | Never | 0 | 0 |
|  | Once or twice a month | 1 | 1 |
|  | Once or twice a week | 2 | 1 |
|  | Every day/almost every day | 3 | 1 |
|  | Missing | 99 | Missing |
|  |  |  |  |
| 3. toothbrushing | Never | 0 | 0 |
|  | Once or twice a month | 1 | 1 |
|  | Once or twice a week | 2 | 1 |
|  | Every day/almost every day | 3 | 1 |
|  | Missing | 99 | Missing |
|  |  |  |  |
| 4. smiling, laughing and showing teeth without embarrassment | Never | 0 | 0 |
|  | Once or twice a month | 1 | 1 |
|  | Once or twice a week | 2 | 1 |
|  | Every day/almost every day | 3 | 1 |
|  | Missing | 99 | Missing |
|  |  |  |  |
| 5. sleeping and relaxing | Never | 0 | 0 |
|  | Once or twice a month | 1 | 1 |
|  | Once or twice a week | 2 | 1 |
|  | Every day/almost every day | 3 | 1 |
|  | Missing | 99 | Missing |
|  |  |  |  |
| 6. emotional balance | Never | 0 | 0 |
|  | Once or twice a month | 1 | 1 |
|  | Once or twice a week | 2 | 1 |
|  | Every day/almost every day | 3 | 1 |
|  | Missing | 99 | Missing |
|  |  |  |  |
| 7. social contact | Never | 0 | 0 |
|  | Once or twice a month | 1 | 1 |
|  | Once or twice a week | 2 | 1 |
|  | Every day/almost every day | 3 | 1 |
|  | Missing | 99 | Missing |
|  |  |  |  |
| 8. schoolwork | Never | 0 | 0 |
|  | Once or twice a month | 1 | 1 |
|  | Once or twice a week | 2 | 1 |
|  | Every day/almost every day | 3 | 1 |
|  | Missing | 99 | Missing |
|  |  |  |  |
