# Supplementary material for: Oral health-related quality of life in 4–16-year-olds with and without juvenile idiopathic arthritis
Source: BMC Oral Health. 2022 Sep 6;22:387. doi: 10.1186/s12903-022-02400-1 (PMC9450232; doi:10.1186/s12903-022-02400-1)
Supplement: Supplementary file 6 — Additional file 6. Description of JIA-specific background variables. [file 12903_2022_2400_MOESM6_ESM.docx]

**Additional file 6**

Previous and on-going medication was registered and categorized into the following groups: 1) no synthetic disease modifying drugs (sDMARDs) nor biologic DMARDs (bDMARDs), 2) sDMARDs, but no bDMARDs, and 3) bDMARDs (with or without sDMARDs). Each group was mutually exclusive. The groups were registered according to ongoing medication, or medication ever used, the last included both previously used and ongoing medication. The participants with JIA were also categorized into groups according to ever used systemic steroid medication. Disease status on the day of visit was recorded according to Wallace et al. (1) and the American College of Rheumatology provisional criteria (2), where inactive disease (on or off medication) included no active arthritis, no fever, no generalized lymphadenopathy, no splenomegaly, no serositis, no rash as a result of JIA, no active uveitis, normal levels of erythrocyte sedimentation rate and C-reactive protein, no morning stiffness exceeding 15 minutes, and MDgloVAS=0. Clinical remission on medication was defined as six continuous months of inactive disease on medications, and remission off medication as twelve continuous months of inactive disease and no anti-rheumatic medication (1). Self-reported physical disability was measured by the validated patient/parent-reported disease-specific childhood health assessment questionnaire (CHAQ) (0=no difficulty and 3=unable to perform), the patient/parent visual analogue scale (VAS) pain (0=no pain and 10=worst possible pain) and overall well-being PRgloVAS (0=best and 10=worst) (3, 4). Global disease activity was registered by the physician using MDgloVAS (0=no activity and 10=maximum activity). VAS pain, PRgloVAS and MDgloVAS were registered on a 21-numbered circle VAS. Patient/parent-reported measures were reported by the parents/proxy if the child were younger than nine years, otherwise by the patient.

**References**

1. Wallace CA, Ruperto N, Giannini E, Childhood A, Rheumatology Research A, Pediatric Rheumatology International Trials O, et al. Preliminary criteria for clinical remission for select categories of juvenile idiopathic arthritis. J Rheumatol. 2004;31(11):2290-4.

2. Wallace CA, Giannini EH, Huang B, Itert L, Ruperto N, Childhood Arthritis Rheumatology Research A, et al. American College of Rheumatology provisional criteria for defining clinical inactive disease in select categories of juvenile idiopathic arthritis. Arthritis Care Res (Hoboken). 2011;63(7):929-36.

3. Ruperto N, Ravelli A, Pistorio A, Malattia C, Cavuto S, Gado-West L, et al. Cross-cultural adaptation and psychometric evaluation of the Childhood Health Assessment Questionnaire (CHAQ) and the Child Health Questionnaire (CHQ) in 32 countries. Review of the general methodology. Clin Exp Rheumatol. 2001;19(4 Suppl 23):S1-9.

4. Selvaag AM, Ruperto N, Asplin L, Rygg M, Landgraf JM, Forre O, et al. The Norwegian version of the Childhood Health Assessment Questionnaire (CHAQ) and the Child Health Questionnaire (CHQ). Clin Exp Rheumatol. 2001;19(4 Suppl 23):S116-20.
