# Supplementary material for: Oral health-related quality of life in 4–16-year-olds with and without juvenile idiopathic arthritis
Source: BMC Oral Health. 2022 Sep 6;22:387. doi: 10.1186/s12903-022-02400-1 (PMC9450232; doi:10.1186/s12903-022-02400-1)
Supplement: Supplementary file 7 — Additional file 7. Table S1. Categories for JIA-specific background variables, as originally coded and re-coded for analyses. [file 12903_2022_2400_MOESM7_ESM.docx]

**Additional file 7**

Table S1. Categories for disease-specific features, as originally coded (if obtained) and re-coded for analyses.

| Variables | Categories | Original code | New code |
| --- | --- | --- | --- |
| JIA category | Systemic arthritis |  | Missing |
|  | Oligoarthritis persistent |  | 1 |
|  | Oligoarthritis extended |  | 2 |
|  | Polyarthritis, RF positive |  | 2 |
|  | Polyarthritis, RF negative |  | 2 |
|  | Psoriatic arthritis |  | 2 |
|  | Enthesitis-related arthritis |  | 2 |
|  | Undifferentiated arthritis |  | Missing |
| Age at JIA onset | 6 years or less |  | 0 |
|  | Over 6 years |  | 1 |
| Duration of the JIA disease | 5 years or less |  | 0 |
|  | Over 5 years |  | 1 |
| Steroids ever used | Steroids ever used |  | 1 |
|  | No steroids ever used |  | 0 |
| DMARDs ongoing | No sDMARDs nor bDMARDs ongoing |  | 0 |
|  | No bDMARDs, but ongoing use of sDMARDs |  | 1 |
|  | No sDMARDs, but ongoing use of bDMARDs or bDMARDs and sDMARDs use ongoing |  | 2 |
| DMARDs ever used | No sDMARDs nor bDMARDs ever used |  | 0 |
|  | No bDMARDs ever used, but sDMARDs have been used |  | 1 |
|  | No sDMARDs ever used, but bDMARDs have been used. Or bDMARDs and sDMARDs have been used |  | 2 |
| Disease status on the day of visit* | Continued activity since onset | 1 | 1 |
|  | Flare | 2 | 1 |
|  | Inactive disease on off medication but not yet remission | 3 | 0 |
|  | Remission on medication | 4 | 0 |
|  | Remission off medication | 5 | 0 |
| MDgloVAS | VAS score=0 |  | 0 |
|  | VAS score>0 |  | 1 |
|  | Missing |  | Missing |
| VAS pain | VAS score=0 |  | 0 |
|  | VAS score>0 |  | 1 |
|  | Missing |  | Missing |
| PRgloVAS | VAS score=0 |  | 0 |
|  | VAS score>0 |  | 1 |
|  | Missing |  | Missing |
| CHAQ items | Without any difficulty | 1 | 0 |
|  | With some difficulty | 2 | 1 |
|  | With much difficulty | 3 | 1 |
|  | Unable to do | 4 | 1 |
|  | Not applicable | 90 | 0 |
|  | Missing * | 99 | 0 |

**Disease activity according to Wallace and the American College of Rheumatology provisional criteria (1, 2).* *RF=rheumatoid factor.* *sDMARDs=synthetic disease-modifying antirheumatic drugs.* *bDMARDs=biologic disease-modifying antirheumatic drugs. MDgloVAS=Physician's global assessment of disease activity visual analogue scale. VAS pain=patient/parent-reported pain intensity visual analogue scale. PRgloVAS=Patient's global assessment of overall wellbeing visual analogue scale. CHAQ=Childhood Health Assessment Questionnaire. * If all questions of a category (8 categories in total) were missing, CHAQ was not calculated.*

**References**

1. Wallace CA, Ruperto N, Giannini E, Childhood A, Rheumatology Research A, Pediatric Rheumatology International Trials O, et al. Preliminary criteria for clinical remission for select categories of juvenile idiopathic arthritis. J Rheumatol. 2004;31(11):2290-4.

2. Wallace CA, Giannini EH, Huang B, Itert L, Ruperto N, Childhood Arthritis Rheumatology Research A, et al. American College of Rheumatology provisional criteria for defining clinical inactive disease in select categories of juvenile idiopathic arthritis. Arthritis Care Res (Hoboken). 2011;63(7):929-36.
