# Supplementary material for: Oral health-related quality of life in 4–16-year-olds with and without juvenile idiopathic arthritis
Source: BMC Oral Health. 2022 Sep 6;22:387. doi: 10.1186/s12903-022-02400-1 (PMC9450232; doi:10.1186/s12903-022-02400-1)
Supplement: Supplementary file 8 — Additional file 8. Table S1. Discriminant validity of the Early Childhood Oral Health Impact Scale (ECOHIS) and Child Oral Impacts on Daily Performances (Child-OIDP) according to the global measures and group affiliation. [file 12903_2022_2400_MOESM8_ESM.docx]

**Additional file 8**

Table S1. Discriminant validity of the Early Childhood Oral Health Impact Scale (ECOHIS) and Child Oral Impacts on Daily Performances (Child-OIDP) according to the global measures and group affiliation.

|  | **ECOHIS** | | | | | | | |
| --- | --- | --- | --- | --- | --- | --- | --- | --- |
|  | Individuals with JIA (n=96) | | | | Controls (n=98) | | | |
|  | **Good oral health**  (n=79)  n (%) | **Poor oral health**  (n=13)  n (%) | **Satisfied with appearance of teeth**  (n=68)  n (%) | **Dissatisfied with appearance of teeth**  (n=24)  n (%) | **Good oral health**  (n=95)  n (%) | **Poor oral health**  (n=3)  n (%) | **Satisfied with appearance of teeth**  (n=89)  n (%) | **Dissatisfied with appearance of teeth**  (n=9)  n (%) |
| **Child impact score>0 *** | 60 (76.0) | 13 (100.0) **^a^** | 51 (75.0) | 22 (91.7) | 62 (65.3) | 2 (66.7) | 55 (61.8) | 9 (100.0) **^a^** |
| **Family impact score>0 *** | 31 (39.2) | 12 (92.3) **^b^** | 26 (38.2) | 17 (70.8) **^a^** | 24 (25.3) | 1 (33.3) | 19 (21.4) | 6 (66.7) **^a^** |
| **ECOHIS total score>0 *** | 63 (79.8) | 13 (100.0) | 53 (77.9) | 23 (95.8) **^a^** | 65 (68.4) | 2 (66.7) | 58 (65.2) | 9 (100.0) **^a^** |
|  |  |  |  |  |  |  |  |  |
|  |  |  |  |  |  |  |  |  |
|  | **Child-OIDP** | | | | | | | |
|  | Individuals with JIA (n=125) | | | | Controls (n=124) | | | |
|  | **Good oral health**  (n=97)  n (%) | **Poor oral health**  (n=27)  n (%) | **Satisfied with appearance of teeth**  (n=87)  n (%) | **Dissatisfied with appearance of teeth**  (n=37)  n (%) | **Good oral health**  (n=107)  n (%) | **Poor oral health**  (n=17)  n (%) | **Satisfied with appearance of teeth**  (n=89)  n (%) | **Dissatisfied with appearance of teeth**  (n=35)  n (%) |
| **Child-OIDP simple count (SC) score>0** | 27 (27.8) | 6 (22.2) | 18 (20.7) | 14 (37.8) **^a^** | 21 (19.6) | 6 (35.3) | 12 (13.5) | 15 (42.9) **^b^** |

***^a^*** *p<0.05; χ2 test.* ***^b^*** *p≤0.001; χ2 test. * The scores are dichotomized as 0 not affected and 1 affected. Some participants did not respond to the global measures.*
