# Supplementary material for: Oral health-related quality of life in 4–16-year-olds with and without juvenile idiopathic arthritis
Source: BMC Oral Health. 2022 Sep 6;22:387. doi: 10.1186/s12903-022-02400-1 (PMC9450232; doi:10.1186/s12903-022-02400-1)
Supplement: Supplementary file 9 — Additional file 9. Table S1. Group affiliation, socio-behavioral, and clinical characteristics in relation to the outcome variable Early Childhood Oral Health Impact Scale (ECOHIS) total score and Child Oral Impacts on Daily Performance (Child-OIDP) simple count (SC) score. Unadjusted and adjusted negative binominal regressions. [file 12903_2022_2400_MOESM9_ESM.docx]

**Additional file 9**

Table S1. Group affiliation, socio-behavioral, and clinical characteristics in relation to the outcome variable Early Childhood Oral Health Impact Scale (ECOHIS) total score and Child Oral Impacts on Daily Performance (Child-OIDP) simple count (SC) score. Unadjusted and adjusted negative binominal regressions.

|  |  | Unadjusted regression | | | | | | Adjusted regression | | | | | |
| --- | --- | --- | --- | --- | --- | --- | --- | --- | --- | --- | --- | --- | --- |
|  |  | ECOHIS total score | | | Child-OIDP SC score | | | ECOHIS total score | | | Child-OIDP SC score | | |
|  |  | IRR | 95% CI | p-value | IRR | 95% CI | p-value | IRR | 95% CI | p-value | IRR | 95% CI | p-value |
| Group affiliation | Control group | ref |  |  | ref |  |  | ref |  |  | ref |  |  |
|  | JIA | 1.61 | (1.15–2.25) | **0.005** | 1.73 | (0.93–3.19) | 0.081 | 1.61 | (1.16–2.23) | **0.004** | 1.88 | (0.88–4.00) | 0.104 |
| Educational level of mother | University/college | ref |  |  | ref |  |  | ref |  |  | ref |  |  |
|  | High school/vocational school | 1.02 | (0.72–1.44) | 0.913 | 0.67 | (0.29–1.53) | 0.341 | 0.81 | (0.58–1.14) | 0.221 | 0.35 | (0.15–0.78) | **0.011** |
| Educational level of father | University/college | ref |  |  | ref |  |  | ref |  |  | ref |  |  |
|  | High school/vocational school | 1.18 | (0.85–1.62) | 0.321 | 0.94 | (0.48–1.83) | 0.852 | 1.16 | (0.83–1.62) | 0.395 | 0.85 | (0.41–1.75) | 0.651 |
| Household structure ^a^ | Two caregivers **^c^** | ref |  |  | ref |  |  |  |  |  |  |  |  |
|  | One caregiver | 1.04 | (0.69–1.58) | 0.851 | 0.96 | (0.47–1.99) | 0.922 |  |  |  |  |  |  |
| Frequency of toothbrushing, n (%) | Twice a day, or more | ref |  |  | ref |  |  | ref |  |  |  |  |  |
|  | Once a day or less/do not know | 1.13 | (0.82–1.57) | 0.457 | 0.86 | (0.40–1.86) | 0.704 | 1.14 | (0.81–1.62) | 0.395 |  |  |  |
| Frequency of tooth flossing during the last 3 months, n (%) | Daily or more | ref |  |  | ref |  |  |  |  |  |  |  |  |
|  | Several times weekly or less/do not know | 0.83 | (0.48–1.41) | 0.486 | 0.70 | (0.26–1.89) | 0.483 |  |  |  |  |  |  |
| During toothbrushing, gingival bleeding occurs | Never | ref |  |  | ref |  |  | ref |  |  | ref |  |  |
|  | Sometimes or more/do not know | 1.38 | (1.02–1.87) | **0.037** | 1.66 | (0.79–3.51) | 0.185 | 1.28 | (0.93–1.76) | 0.128 | 1.42 | (0.63–3.22) | 0.399 |
| During toothbrushing, pain or discomfort occurs | No | ref |  |  | ref |  |  |  |  |  | ref |  |  |
|  | Yes/do not know | 1.46 | (0.84–2.54) | 0.179 | 4.34 | (2.30–8.21) | **<0.001** |  |  |  | 5.23 | (2.58–10.60) | **<0.001** |
| Dental caries *** | d_1-5_ft/D_1-5_FT **^b^**=0 | ref |  |  | ref |  |  | ref |  |  | ref |  |  |
|  | d_1-5_ft/D_1-5_FT **^b^**>0 | 1.61 | (1.15–2.24) | **0.005** | 1.23 | (0.61–2.45) | 0.564 | 1.68 | (1.22–2.31) | **0.001** | 1.05 | (0.49–2.26) | 0.903 |

***^a^*** *Regressed on group affiliation and adjusted for socio-behavioral factors (parental educational level and bleeding during toothbrushing) and dental caries.*  ***^b^*** *Also includes living across two households, given two caregivers in both households.* ***^c^*** *Decayed and/or filled teeth in the primary or permanent dentition, enamel caries included. OR=odds ratios. CI=confidence interval. IRR=incidence rate ratios. JIA=juvenile idiopathic arthritis.*
