# Supplementary material for: Oral health-related quality of life in 4–16-year-olds with and without juvenile idiopathic arthritis
Source: BMC Oral Health. 2022 Sep 6;22:387. doi: 10.1186/s12903-022-02400-1 (PMC9450232; doi:10.1186/s12903-022-02400-1)
Supplement: Supplementary file 10 — Additional file 10. Table S1. Disease-specific features and dental caries in relation to the outcome variable Early Childhood Oral Health Impact Scale (ECOHIS) total score and Child Oral Impacts on Daily Performance (Child-OIDP) simple count (SC) score. Unadjusted and adjusted negative binominal regressions. [file 12903_2022_2400_MOESM10_ESM.docx]

**Additional file 10**

Table S1. Disease-specific features and dental caries in relation to the outcome variable Early Childhood Oral Health Impact Scale (ECOHIS) total score and Child Oral Impacts on Daily Performance (Child-OIDP) simple count (SC) score. Unadjusted and adjusted negative binominal regressions.

|  |  | ECOHIS total score | | | | | | | Child-OIDP simple count (SC) score | | | | | | |
| --- | --- | --- | --- | --- | --- | --- | --- | --- | --- | --- | --- | --- | --- | --- | --- |
|  |  |  | Unadjusted regressions | | | Adjusted regressions **^a^** | | |  | Unadjusted regressions | | | Adjusted regressions **^a^** | | |
|  |  | n | IRR | 95% CI | p-value | IRR | 95% CI | p-value | n | IRR | 95% CI | p-value | IRR | 95% CI | p-value |
| JIA category | Oligoarthritis persistent | 32 | ref |  |  | ref |  |  | 44 | ref |  |  | ref |  |  |
|  | Not oligoarthritis persistent **^b^** | 45 | 1.71 | (1.03–2.83) | **0.038** | 1.50 | (0.89–2.54) | 0.129 | 62 | 9.51 | (3.22–28.13) | **<0.001** | 10.96 | (3.87–31.04) | **<0.001** |
| Age at JIA onset | ≤6 years | 64 | ref |  |  | ref |  |  | 46 | ref |  |  | ref |  |  |
|  | >6 years | 32 | 0.86 | (0.55–1.34) | 0.509 | 0.74 | (0.45–1.22) | 0.235 | 79 | 1.05 | (0.49–2.26) | 0.897 | 1.31 | (0.51–3.33) | 0.573 |
| Disease duration | ≤5 years | 57 | ref |  |  | ref |  |  | 60 | ref |  |  | ref |  |  |
|  | >5 years | 39 | 1.55 | (1.04–2.31) | **0.032** | 1.58 | (1.05–2.36) | **0.027** | 65 | 1.14 | (0.53–2.43) | 0.742 | 1.36 | (0.58–3.18) | 0.476 |
| Steroids, ever used | No steroids ever used | 74 | ref |  |  | ref |  |  | 99 | ref |  |  | ref |  |  |
|  | Steroids ever used | 22 | 1.28 | (0.78–2.08) | 0.326 | 1.17 | (0.70–1.94) | 0.545 | 26 | 1.62 | (0.68–3.85) | 0.273 | 2.87 | (0.99–8.32) | 0.052 |
| DMARDs, ongoing | No sDMARDs nor bDMARDs ongoing | 26 | ref |  |  | ref |  |  | 49 | ref |  |  | ref |  |  |
|  |  |  |  |  |  |  |  |  |  |  |  |  |  |  |  |
|  | sDMARDs, but no bDMARDs ongoing | 34 | 1.40 | (0.75–2.63) | 0.293 | 1.45 | (0.83–2.53) | 0.189 | 27 | 2.12 | (0.83–5.43) | 0.119 | 1.63 | (0.56–4.78) | 0.371 |
|  |  |  |  |  |  |  |  |  |  |  |  |  |  |  |  |
|  | bDMARDs ongoing **^c^** | 36 | 1.34 | (0.73–2.45) | 0.349 | 1.41 | (0.79–2.50) | 0.246 | 49 | 1.46 | (0.53–3.99) | 0.462 | 1.79 | (0.65–4.89) | 0.260 |
| DMARDs, ever used | No sDMARDs nor bDMARDs ever used | 15 |  |  |  |  |  |  | 37 |  |  |  |  |  |  |
|  |  |  |  |  |  |  |  |  |  |  |  |  |  |  |  |
|  | sDMARDs, but no bDMARDs ever used | 43 | 1.40 | (0.59–3.32) | 0.452 | 1.55 | (0.69–3.46) | 0.288 | 38 | 1.84 | (0.63–5.36) | 0.265 | 1.20 | (0.40–3.63) | 0.750 |
|  |  |  |  |  |  |  |  |  |  |  |  |  |  |  |  |
|  | bDMARDs ever used **^c^** | 38 | 1.32 | (0.56–3.14) | 0.525 | 1.51 | (0.64–3.55) | 0.346 | 50 | 1.44 | (0.46–4.51) | 0.533 | 1.61 | (0.54–4.81) | 0.397 |
| Disease status ^d^ | Inactive disease/  remission  on/off medication | 59 | ref |  |  | ref |  |  | 74 | ref |  |  | ref |  |  |
|  |  |  |  |  |  |  |  |  |  |  |  |  |  |  |  |
|  | Continued activity/flare | 37 | 1.07 | (0.71–1.62) | 0.753 | 1.07 | (0.72–1.59) | 0.727 | 51 | 2.90 | (1.28–6.57) | **0.011** | 2.48 | (0.99–6.25) | 0.054 |
| MDgloVAS | VAS=0 | 63 | ref |  |  | ref |  |  | 79 | ref |  |  | ref |  |  |
|  | VAS>0 | 33 | 1.13 | (0.74–1.73) | 0.565 | 1.08 | (0.72–1.63) | 0.710 | 46 | 2.68 | (1.24–5.77) | **0.012** | 2.05 | (0.84–5.04) | 0.117 |
| VAS pain | VAS=0 | 48 | ref |  |  | ref |  |  | 64 | ref |  |  | ref |  |  |
|  | VAS>0 | 48 | 1.63 | (1.03–2.61) | **0.039** | 1.52 | (0.96–2.41) | 0.077 | 57 | 6.01 | (2.09–17.23) | **0.001** | 6.77 | (1.86–24.68) | **0.004** |
| PRgloVAS | VAS=0 | 25 | ref |  |  | ref |  |  | 34 | ref |  |  | ref |  |  |
|  | VAS>0 | 71 | 1.70 | (0.94–3.10) | 0.082 | 1.56 | (0.89–2.74) | 0.121 | 87 | 3.42 | (1.11–10.57) | **0.033** | 2.05 | (0.53–8.01) | 0.301 |
| CHAQ ^e^ | CHAQ=0 | 37 | ref |  |  | ref |  |  | 54 | ref |  |  | ref |  |  |
|  | CHAQ>0 | 59 | 1.18 | (0.76–1.83) | 0.450 | 1.25 | (0.81–1.95) | 0.319 | 71 | 6.59 | (2.44–17.80) | **<0.001** | 5.60 | (1.92–16.37) | **0.002** |
| Dental caries | d_1-5_ft/D_1-5_FT **^f^**=0 | 65 | ref |  |  | ref |  |  | 54 | ref |  |  | ref |  |  |
|  | d_1-5_ft/D_1-5_FT **^f^**>0 | 29 | 1.57 | (1.06–2.32) | **0.025** | 1.48 | (0.98–2.23) | 0.063 | 68 | 1.09 | (0.45–2.62) | 0.854 | 0.44 | (0.14–1.42) | 0.170 |

***^a^*** *Adjusted for: gender, age and educational level of mother and educational level of father.* ***^b^*** *Includes oligoarthritis extended, polyarthritis RF positive and RF negative, psoriatic arthritis, and enthesitis-related arthritis* ***^c^*** *With or without sDMARDs.* ***^d^*** *Disease activity according to Wallace (1) and the American College of Rheumatology provisional criteria (2).* ***^e^*** *Self-reported physical disability measured with the disease-specific and validated Childhood Health Assessment Questionnaire (CHAQ) (0=no difficulty and 3=unable to do)(3).* ***^f^*** *Decayed and/or filled teeth in the primary or permanent dentition, enamel caries included. Some registrations are missing. * Oligoarthritis extended (n=11), polyarthritis, RF positive (n=1) and RF negative (n=24), psoriatic arthritis (n=3), and enthesitis-related arthritis (n=6). ** Oligoarthritis extended (n=11), polyarthritis, RF positive (n=1) and RF negative (n=26), psoriatic arthritis (n=5), and enthesitis-related arthritis (n=17). RF=rheumatoid factor. sDMARDs=synthetic disease-modifying antirheumatic drugs. bDMARDs=biologic disease-modifying antirheumatic drugs. MDgloVAS=physician's global assessment of disease activity visual analogue scale (VAS). VAS pain=patient/parent-reported pain intensity VAS. PRgloVAS=patient's global assessment of overall wellbeing VAS. CHAQ=Childhood Health Assessment Questionnaire. OR=odds ratios. CI=confidence interval. IRR=incidence rate ratios. JIA=juvenile idiopathic arthritis.*

**References**

1. Wallace CA, Ruperto N, Giannini E, Childhood A, Rheumatology Research A, Pediatric Rheumatology International Trials O, et al. Preliminary criteria for clinical remission for select categories of juvenile idiopathic arthritis. J Rheumatol. 2004;31(11):2290-4.

2. Wallace CA, Giannini EH, Huang B, Itert L, Ruperto N, Childhood Arthritis Rheumatology Research A, et al. American College of Rheumatology provisional criteria for defining clinical inactive disease in select categories of juvenile idiopathic arthritis. Arthritis Care Res (Hoboken). 2011;63(7):929-36.

3. Ruperto N, Ravelli A, Pistorio A, Malattia C, Cavuto S, Gado-West L, et al. Cross-cultural adaptation and psychometric evaluation of the Childhood Health Assessment Questionnaire (CHAQ) and the Child Health Questionnaire (CHQ) in 32 countries. Review of the general methodology. Clin Exp Rheumatol. 2001;19(4 Suppl 23):S1-9.
